# Radiomic Pipelines for Prostate Cancer in External Beam Radiation Therapy: A Review of Methods and Future Directions

**DOI:** 10.3390/jcm13133907

**Published:** 2024-07-03

**Authors:** Bruno Mendes, Inês Domingues, João Santos

**Affiliations:** 1 Research Center of the Portuguese Institute of Oncology of Porto (CI-IPOP), Medical Physics, Radiobiology and Radiological Protection Group, R. Dr. António Bernardino de Almeida, 4200-072 Porto, Portugal; inesdomingues@gmail.com (I.D.); j.a.miranda.santos@gmail.com (J.S.); 2 Faculty of Engineering of the University of Porto (FEUP), R. Dr. Roberto Frias, 4200-465 Porto, Portugal; 3 Polytechnic Institute of Coimbra, Coimbra Institute of Engineering, Rua Pedro Nunes-Quinta da Nora, 3030-199 Coimbra, Portugal; 4 School of Medicine and Biomedical Sciences (ICBAS), R. Jorge de Viterbo Ferreira 228, 4050-313 Porto, Portugal

**Keywords:** radiomics, prostate cancer, machine learning, review

## Abstract

**Background**: Prostate Cancer (PCa) is asymptomatic at an early stage and often painless, requiring only active surveillance. External Beam Radiotherapy (EBRT) is currently a curative option for localised and locally advanced diseases and a palliative option for metastatic low-volume disease. Although highly effective, especially in a hypofractionation scheme, 17.4% to 39.4% of all patients suffer from cancer recurrence after EBRT. But, radiographic findings also correlate with significant differences in protein expression patterns. In the PCa EBRT workflow, several imaging modalities are available for grading, staging and contouring. Using image data characterisation algorithms (radiomics), one can provide a quantitative analysis of prognostic and predictive treatment outcomes. **Methods**: This literature review searched for original studies in radiomics for PCa in the context of EBRT. Following the Preferred Reporting Items for Systematic Reviews and Meta-Analyses (PRISMA) guidelines, this review includes 73 new studies and analyses datasets, imaging modality, segmentation technique, feature extraction, selection and model building methods. **Results**: Magnetic Resonance Imaging (MRI) is the preferred imaging modality for radiomic studies in PCa but Computed Tomography (CT), Positron Emission Tomography (PET) and Ultrasound (US) may offer valuable insights on tumour characterisation and treatment response prediction. **Conclusions**: Most radiomic studies used small, homogeneous and private datasets lacking external validation and variability. Future research should focus on collaborative efforts to create large, multicentric datasets and develop standardised methodologies, ensuring the full potential of radiomics in clinical practice.

## 1. Introduction

External Beam Radiotherapy (EBRT) is a curative option for localised and locally advanced disease and a palliative option for metastatic low-volume disease [1,2]. Three-dimensional images provided the adoption of 3D-conformal radiation therapy in a clinical setting, while advances in multileaf collimators allowed the introduction of Intensity Modulated Radiation Therapy (IMRT), improving target coverage and reducing Organs At Risk (OARs) dose [3]. Prostate Cancer (PCa) benefits from high daily fraction doses (hypofractionation), presenting the same clinical outcome as prostatectomy [3]. Still, there is a 17.4% biochemical recurrence rate at 5 years and 39.4% at 10 years for patients undergoing primary EBRT [4]. Currently, the trigger biomarker is the Prostate Specific Antigen (PSA) level rise, which may not necessarily mean a metastatic progression or a change in survival endpoints. Whether recurrence happens locally or at distant sites, prediction also depends on the initial risk grouping and pre- and post-treatment factors [1]. Additionally, PCa is a heterogeneous disease at the pathological, molecular, genetic and clinical levels, with 10-year survival rates varying with relapse [1].

Heterogeneous solid cancers may limit invasive biopsies but open an opportunity for medical imaging. In the EBRT workflow, patients usually undergo a Computed Tomography (CT) scan, providing the anatomical basics for treatment planning and attenuation coefficients for dose estimations. In this stage, experts define tumour and tissue-related volumes aided by Magnetic Resonance Imaging (MRI) and Positron Emission Tomography (PET) using 18F or 68Ga if available [5], presenting improved soft-tissue contrast. Also, CT images have a higher spatial resolution than MRI, allowing the evaluation of density, shape and texture characteristics. Radiographic findings also correlate with significant differences in protein expression patterns [6]. In this context, extracting features from radiographic images using data characterisation algorithms (radiomics) may provide a valuable tool for PCa evaluation during EBRT. The hypothesis behind radiomics is that quantitative analysis of medical images may have a similar prognosis power to phenotypes and gene protein signatures. The potential of radiomics to provide intel on the intensity, shape, size or tumour volume is distinct from clinical reports, biopsies or genomic studies. When correlated with clinical outcomes, radiomics can be used as evidence-based clinical decision support systems [7]. In the EBRT workflow, there are several potential sources of radiomic data yet unexplored in a clinical context. Digital images such as CT, PET, MRI, Cone Beam Computed Tomography (CBCT) or Ultrasound are usually available for every PCa patient. The interpretation of these images can, in the future, be augmented with radiomic data, potentially aiding in detection, diagnosis, prognosis, treatment responses and disease monitoring [7].

**Figure 1:**
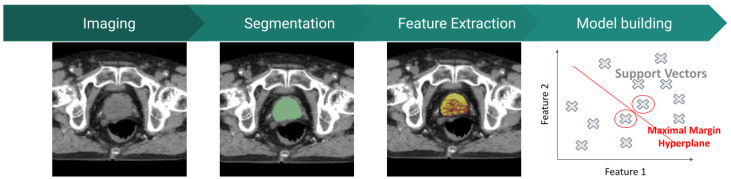
Radiomics workflow.

Radiomic studies usually follow a discrete pipeline, involving several steps such as image acquisition, segmentation, feature extraction and model building [6]. As an emerging field in medicine, researchers use several methodologies for each one of the steps. This work intends to be an extensive literature review of the current advanced mathematical methods used in radiomic studies, especially concerning the potential applicability in the EBRT workflow for PCa. Radiomics interest is rapidly growing but lacks procedural benchmarking. This review may enlighten the potential of radiomic features and enhance their limitations and future improvements. Figure 1 shows the workflow for radiomic studies as initially proposed by Lambin et al. [6].

Following this introduction, Section 2 presents the used methodology and obtained results. The analysis follows the different steps in a radiomics pipeline framed in a PCa EBRT context. We begin by analysing the size of the datasets used by authors in their research, followed by the imaging modalities, segmentation methods, feature extraction and commonly used techniques for model building. The best obtained results and built models are presented in Section 2.6. Finally, Section 3 presents the final remarks, conclusions and main contributions.

## 2. Methodology and Results

This literature review used the available databases from Scopus, PubMed (NIM) and ScienceDirect with the following search terms: Radiomics AND Prostate AND Cancer AND Machine AND Learning AND (Staging OR Grading OR Prediction OR Aggressiveness) within the article title, abstract and keywords. The search was performed following the Preferred Reporting Items for Systematic Reviews and Meta-Analyses (PRISMA) guidelines [8] including original research articles published from 2019 to 2023. Data extraction included article title, year and author, population description with inclusion and exclusion criteria, number of patients used in the cohort with age range, imaging modality and settings, segmentation method, number of features extracted and types, preprocessing methods, feature selection methods, statistical/machine learning techniques to build the models, and evaluation metrics. An outcome table was also built for the best obtained models. The analysis and data extraction was performed using the Covidence platform [9].

**Figure 2:**
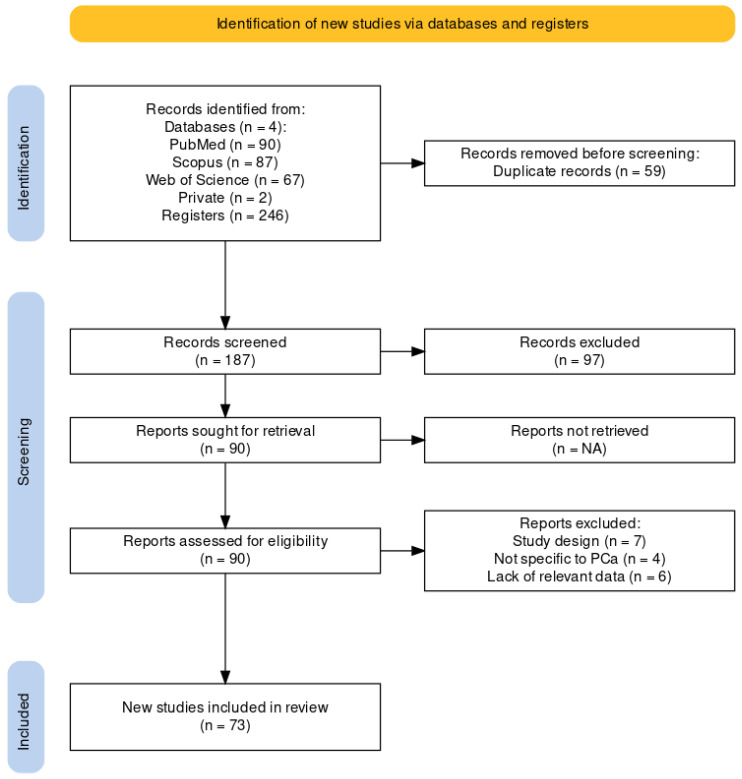
PRISMA Diagram. Generated by Haddaway et al. [10].

The search outputted 246 unique results after the removal of 59 duplicate entries using the Covidence automated tool [9]. The title and abstract of the remaining 187 studies were carefully analysed. In total, 47 were reviews, overviews or surveys and not original work. Fifty were not specifically related to PCa or radiomics and therefore not included. A total of 90 reports were eligible for this review. From those, seven had a study design flow not compatible for comparison with other studies, four were not specifically related to PCa and six lacked the data relevant for this review. Following the PRISMA guidelines, 73 studies were included. Figure 2 summarises the followed methodology.

The following sections present an analysis of the selected studies. starting with the datasets used, the imaging modalities, the segmentation methods, the radiomic features and the machine learning models following the radiomics pipeline proposed by Lambin et al. [6]. Section 2.2 presents a brief summary of each study, grouped by imaging modality and ordered by year of publication. Section 2.3 refers to some of the most used segmentation methods found in this review. Section 2.1 analyses the used datasets concerning country and number of patients included in each cohort. Section 2.4 mentions the most used feature extraction methods. Section 2.5 refers to the most used feature selection and model building techniques and, finally, Section 2.6 presents the results obtained by researchers, grouped by imaging modality and ordered by year of publication.

### 2.1. Datasets

**Figure 3:**
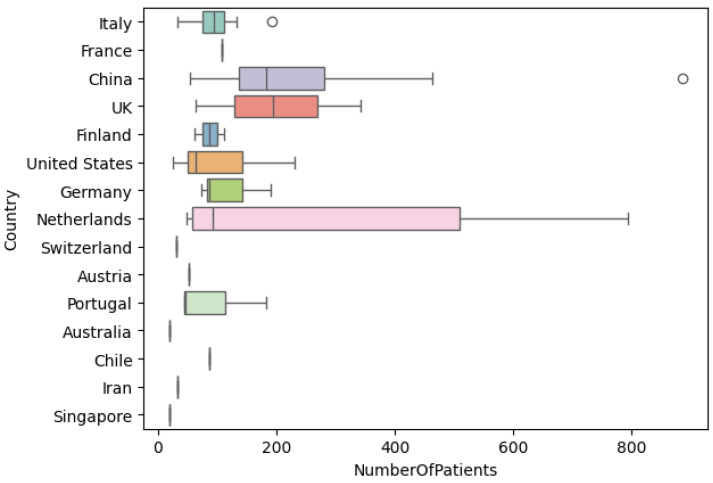
Number of patients distribution per country.

Overall, the mean number of patients included in the reviewed studies is 168. The smallest dataset, with only 19 patients, is from Chan et al. [11] while the largest, with 886 patients, is from Yang et al. [12]. Additionally, only Rodrigues et al. [13] used a public dataset, available in the PROSTATEx challenge. Figure 3 shows the number of patients used in the reviewed studies grouped by country.

### 2.2. Imaging

The available medical imaging systems can obtain high-quality images and thus allow for the the extraction of more informative features. In this context, radiomics may reveal unseen information for PCa and provide a more accurate framework for PCa staging and grading. Recently, Woźnicki et al. [14] showed that adding radiomic features to Prostate Imaging Reporting and Data System (PIRADS) and clinical parameters had superior performance. The quality of extracted features has a strong relationship with the imaging modality used for acquisition.

#### 2.2.1. Ultrasound

Ultrasound (US) is a non-invasive, low-cost, real-time imaging modality that is widely available. It is used for PCa screening, diagnosis, staging, and treatment monitoring. Also, Transrectal Ultrasound Guided Biopsy (TRUS) biopsy guidance is the most common biopsy technique for PCa diagnosis. With TRUS, there is a high risk of under- or oversampling that may lead to overtreatment and/or overdiagnosis. To assess this issue, Wildeboer et al. [15] described the quantification of 3D dynamic contrast-enhanced ultrasound to provide better insights into the TRUS procedure. They extracted radiomic features, attempting to predict TRUS outcomes. More recently, Liang et al. [16] developed a nomogram model based on radiomics and clinical factors associated with a Prostate Cancer diagnosis to distinguish between prostate lesions. The study analysed 128 patients retrospectively, and the radiomics-based model improved diagnostic efficiency and clinical net benefit. The research highlights the importance of radiomics in converting medical features from images into high-dimensional data for better clinical decision-making. In a similar study, Sun et al. [17] used machine learning models from US to improve PCa detection in the peripheral zone. The results showed a promising diagnostic tool aiming to improve the detection of PCa in the peripheral zone using advanced imaging techniques and machine learning models. Using US video clips, Wang et al. [18] developed prediction models showing promising diagnostic efficiency and recently, Qi et al. [19] compared machine learning models using US videos and multi-parametric Magnetic Resonance Imaging (mpMRI) for predicting PCa, with the Support Vector Machine (SVM) model performing best in both the US and MRI groups.

#### 2.2.2. Positron Emission Tomography

With the addition of a radio-tracer, Fluorodeoxyglucose (F-18 FDG), PET became an important tool for diagnosis, staging, grading and assessing the pathological responses of some types of cancers. Mainly used in conjunction with CT or MRI, PET has become a valuable source of radiomics insights. In this review, 11 papers related to PCa were found.

The first study in this review is from Merisaari et al. [20], examining the accuracy of radiomics and machine learning for Diffusion-weighted Imaging (DWI) in 112 patients with PCa. The research found that surface-to-volume ratio and corner detectors are promising techniques for prostate DWI, along with shape features and kurtosis in classification, emphasising the importance of accurate and repeatable radiomic features for effective Prostate Cancer management.

Alongi et al. [21] evaluated tumour heterogeneity with radiomic features from 18F-Cho-PET/CT (18F-Cho is another radio-tracer commonly used). With 94 high-risk PCa patients, they built a model using Discriminant Analysis (DA) evaluating a radiomic signature for each specific Risk Group (RG), predicting disease progression. Cysouw et al. [22] assessed several clinical parameters, like lymph node involvement, metastasis, Gleason Score (GS) and extra-capsular extension, using a Random Forest (RF) model, improving the performance using PET-specific features as the Standardized Uptake Value (SUV). Erle et al. [23] evaluated supervised machine learning models with radiomic features obtained from pre-therapeutic [68Ga]–Prostate-Specific Membrane Antigen (PSMA)–PET–CT patients, showing a significant improvement in identifying pathological uptake in patients with advance PCa. Using the same imaging modality, Moazemi et al. [24] showed potential for predicting responses to 177Lu-PSMA treatment. The radiomics features that best correlate with δPSA are shown to outperform clinical parameters in predicting therapy response. In the same line, Papp et al. [25] proved that [68Ga]Ga-PSMA-11 PET/MRI, when combined with radiomics and machine learning, can provide accurate lesion characterisation and risk prediction for patients with primary PCa without the need for invasive biopsy before surgery.

Later, Pirrone et al. [26] studied the use of a machine learning model to predict treatment response for PCa patients who had undergone re-irradiation. The approach could be useful in tumour dose painting. The dosimetric-based classifier predicts therapy response three years after primary prostate radiation therapy. Yao et al. [27] concluded that SUVmax radiomic features models showed the best predictive performance for PCa.

More recently, Chan et al. [11] used radiomic features in PSMA PET and mpMRI to detect localised Prostate Cancer. The study assesses their value at a voxel level and investigates machine learning models to predict tumour location and grade, suggesting that combining PET and mpMRI radiomic features is better for predicting tumour location than using features from either modality alone. Luining et al. [28] also presented a study using machine learning models to predict high-risk tumour features in patients with PCa with interesting outcomes. Finally, Nai et al. [29] compared quantitative parameters and radiomic features as inputs for machine learning models to predict the GS of PCa lesions. The models evaluated performance with different combinations of inputs and risk factors, with K-nearest Neighbors (KNN) providing the highest accuracy. The study concluded that the combination of inputs and risk factors is crucial in improving the classification performance of machine learning in predicting clinically significant Prostate Cancer lesions.

#### 2.2.3. Magnetic Resonance Imaging

MRI provides superior soft-tissue contrast resolution when compared to other imaging modalities. Tissue properties do not correlate well with signal intensities, but using dynamic contrast-enhanced imaging and the combination of T1- and T2-weighted sequences (mpMRI) overcomes this issue. It is the selected imaging modality by PIRADS to assess several clinical outcomes.

Abdollahi et al. [30] gave the first step towards a personalised diagnosis and treatment of Prostate Cancer, predicting the response of IMRT. Considering high-risk patients that underwent radical prostatectomy, Bourbonne et al. [31] built a radiomics model predictive of Biochemical Recurrence (BCR). Chen et al. [32] studied the use of radiomics-based feature extraction and machine learning to differentiate between malignant and benign prostate tissue, evaluating the aggressiveness of PCa. The study found that the T2WI or Apparent Diffusion Coefficient (ADC) radiomics-based models demonstrated high diagnostic performance, slightly increasing the comprehensive diagnostic efficacy and showing to be more efficient than PIRADS scores in diagnosing PCa. Min et al. [33] developed a radiomic signature from mpMRI to distinguish between clinically significant PCa and clinically insignificant PCa. Parra et al. [34] developed a method to accurately identify Dynamic Contrast Enhanced (DCE) perfusion regions that can assess DCE characteristics related to clinically significant cancers. Toivonen et al. [35] built a model from T2W and ADC with the kurtosis feature and showed improvements in PCa characterisation when compared to models built with DWI. With an independent validation set, Varghese et al. [36] also outperformed PIRADS using mpMRI.

Algohary et al. [37], continuing their previous work, combined peritumoural and intratumoural features in an attempt to stratify PCa patients according to their RG. The results suggest that peritumoural features are more predictive of high-risk PCa. Bernatz et al. [38] evaluated several machine learning algorithms to predict clinically significant PCa. The model obtained with ADC maps performed best. Bleker et al. [39] performed an automatic segmentation approach with an auto-fixed-size Region Of Interest (ROI) for feature extraction, being able to quantify clinically significant PCa with features from the peripheral zone. With a semi-automatic prostate segmentation approach and aiming at PCa BCR prediction, Bourbonne et al. [40] obtained good results. The T2W sequence and ADC maps do provide valuable radiomic data, although the authors refer to different follow-up times and MRI scanner variability and no PIRADS implementation for DCE. Later, Hou et al. [41] provided a model directed to the selection of prostate biopsy candidates using T2W, DWI and ADC with promising results although institution-specific. Li et al. [42] included clinical risk factors together with radiomic features to improve the accuracy of predicting PCa aggressiveness, constructing a nomogram to offer a precise method to predict clinically significant PCa. Woźnicki et al. [14] also combined several clinical outcomes in a PCa evaluation framework.

The introduction of clinical outcomes, as PSA or GS, was referred to by Bevilacqua et al. [43] when assessing the *DWI*_*b*200_ sequence as an issue and confirming the ADC as the leading sequence for detection. Bertelli et al. [44] and Castillo T et al. [45] included Deep Learning features in their frameworks. Both groups concluded that a radiomics model trained on radiologist-provided segmentations is more accurate and generalisable for significant PCa classification than a fully automated deep learning model. Improving their work, Castillo T et al. [46] tested the developed radiomics model on a multi-centre cohort, outperforming the radiologists. The predictive power of radiomics for Extraprostatic Extension (EPE) was assessed by Cuocolo et al. [47]. With a cohort of 193 patients, the study reinforced the combination of radiomic features and machine learning methods for EPE prediction. Li et al. [48] created a personalised, easy-to-use radiomics nomogram tool with potential clinical use in differentiating between clinically significant PCa and clinically insignificant PCa in PIRADS 3 lesions using mpMRI. Peng et al. [49] also evaluated clinically significant PCa and suggested that RF and Linear Regression (LR) models perform better with ADC texture-related parameters in line with the findings from Zhang et al. [50]. Rodrigues et al. [13] performed a study using biparametric MRI (bmMRI), confirming the validity of MRI-based radiomic features and obtaining higher performances with the whole prostate gland.

Algohary et al. [51] continued their work and, in a pilot study, evaluated the longitudinal change in patients treated with MRI-guided lattice extreme ablative dose-boost radiotherapy. The obtained quantitative model showed promise as a predictor of treatment failure. Fan et al. [52] linked radiomics features of mpMRI with five biological traits of PCa, predicting critical biological characteristics of PCa with high accuracy. Gaudiano et al. [53] integrated traditional mpMRI with radiomic features using machine learning models to distinguish between clinically significant PCa and clinically insignificant PCa, and, in line with other studies, also concluded that ADC-based radiomics may provide a valuable tool for characterising PCa lesions. Targeting patients with PSA levels between 4 and 10 ng/mL, Lu et al. [54] combined T2Wi and ADC maps in a radiomic nomogram. Gresser et al. [55] reported the variability of classification performance across several model configurations, and Jing et al. [56] also proposed a nomogram model combining a radiomics signature, obtained from pre-operative bmMRI and the whole prostate gland, with PIRADS. In a different approach, Liu et al. [57] attempted to predict the *P*504*s*/*P*63 immunohistochemical expression with MRI-based radiomics. With an RF classifier, they were able to successfully and non-invasively diagnose PCa. Ma et al. [58] used texture analysis to distinguish benign from malignant lesions in PCa with promising results on patients with a PIRADS 4/5 score. With an interesting approach, Sushentsev et al. [59] used delta-radiomics in active surveillance patients to monitor disease progression. MRI-derived delta-radiomics performs comparably to expert prostate MRI readers in predicting histopathological progression of PCa in active surveillance patients, offering an objective and standardised approach. With an external validation set to predict intraductal carcinoma of the prostate, Yang et al. [12] outperformed the clinical model.

Dominguez et al. [60] developed a model to detect clinically significant PCa based on GS using parametric MRI radiomic features and clinical information, outperforming PIRADS and PSA density in accurately classifying clinically significant PCa. Gaudiano et al. [61] explored the potential of mpMRI as a standalone tool for the early and non-invasive detection of PCa in a selected cohort of PIRADS 3 lesions, obtaining promising results. Isaksson et al. [62] compared the performance of different models for tabular data in a typical radiomics setting while predicting nine different PCa pathology outcomes. The study investigated if multitask learning improves the performance of these models, but the results did not provide a consistent improvement. Jamshidi et al. [63] evaluated radiomics models for diagnosing PCa using high-resolution T2-weighted and DCE MRI, providing a promising method for the reliable detection of prostate lesions in MRI images through the use of a fused model. Jin et al. [64] developed radiomic models from different MRI sequences to distinguish between benign and malignant PIRADS 3 lesions, aiming to cross-validate their generalisation ability across institutions. The results showed a statistically significant difference in PSAD between PCa and benign lesions. Li et al. [65] compared MRI visible and MRI-negative clinically significant PCa using bmMRI radiomic features. The results showed that bmMRI had the highest accuracy to RMRI+ and PIRADS in detecting clinically significant PCa. In another study, Li et al. [66] aimed to differentiate between PCa and Benign Prostatic Hyperplasia (BPH) using an imaging model based on mpMRI images. Liu [67] aimed to evaluate the MRI radiomics models effectiveness in predicting PCa invasion. The radiomics model had similar predictive performance to the clinical radiomics model and better than the single GS. Midya et al. [68] measured radiomic changes in PCa progression on serial MRI scans of patients on active surveillance and examined their correlation with pathologic progression on biopsy. Delta-radiomics had a stronger correlation with upgrade events in comparison to PIRADS and other clinical variables. Prata et al. [69] developed a radiomic tool to predict clinically significant PCa, combining three orthogonal planes with clinical features to assess PCa aggressiveness with promising results. Qiao et al. [70] found that radiomics-based models using mpMRI can identify aggressive PCa, providing non-invasive, synchronous, and objective guidance for clinical decision-making. Qiu et al. [71] used peritumoural radiomics features to predict Grade Group (GG) in PCa patients. Rodrigues et al. [72] compared different segmentation approaches and found that training with heterogeneous data led to the most robust classifiers, while removing features with low InterCorrelation Coefficient (ICC) resulted in the highest generalisation error. A hybrid model combining a selected radiomics dataset with deep features extracted from neural networks was created, but it was found to be less effective than the radiomics model. Stoyanova et al. [73] were the first to demonstrate a machine learning radiomics-based model’s ability to predict patient risk using combined clinical–genomic classification. van den Berg et al. [74] showed that radiomics models can predict EPE in PCa patients on MRI. Xue et al. [75] investigated the impact of random features on MRI radiomics feature selection, modelling, and performance for Prostate Cancer diagnosis. Random features were added progressively, and three feature selection algorithms and two classifiers were used to build the models. They found that including additional random features may slightly impact feature selection, but it does not have a substantial impact on MRI radiomics model performance. Zhao et al. [76] aimed to predict clinically significant PCa in PIRADS 3 lesions located in the transition zone, with the XGboost model found to be the most effective approach for this purpose. Zhong et al. [77] developed a model using radiomic features from whole-prostate MRI to predict tumour hypoxia before radiotherapy to help optimise individualised treatment. Finally, Zhou et al. [78] developed MRI-based radiomics signatures to predict Ki-67 expression status and GS, showing that both are associated with poor patient survival.

#### 2.2.4. Computed Tomography

CT images have a high spatial resolution allowing the evaluation of density, shape and texture characteristics. Although CT images lack characteristic manifestation [27] and seem to be a poor candidate for radiomic feature extraction, their use for volume delineation in the treatment planning stage makes them always available. Also, they are used for PCa in the staging for metastases in intermediate- and high-risk patients [2]. From the selected papers in this review, only two evaluated the value of radiomic data from CT imaging both for patient risk stratification based on the histologically obtained GS.

Osman et al. [79] presented a study exploring the use of CT-based radiomics features, showing promising results in identifying low GS from high GS and low-risk from high-risk patients. The study also includes a test–retest analysis to investigate the robustness and stability of the extracted features.

Later, Mendes et al. [80] focused on the use of CT images in predicting the aggressiveness of PCa. They discuss the challenges involved in diagnosing and grading prostate cancer, the role of EBRT, and the potential of radiomics in assessing PCa aggressiveness. The study shows that finding a radiomic signature to predict PCa aggressiveness is challenging when using CT images, providing however a research baseline to contribute to the ongoing optimisation of PCa treatment and decision outcomes.

#### 2.2.5. Cone Beam Computed Tomography

Two of the main limiting factors in the effectiveness of EBRT in PCa are patient position and internal organ variations. Currently, onboard CBCT imaging overcomes this issue, allowing the verification and adjustment of the treatment target by overlaying the initial CT scan and delineated contours. However, this is suboptimal due to the low soft-tissue contrast presented by both CT and CBCT [81].

In a pioneer study, Bosetti et al. [82] examined the usefulness of CBCT-based radiomics in PCa involving 31 patients, and radiomics features from weekly CBCT scans. LR models were developed for tumour stage, GS, PSA level, and risk stratification, as well as for predicting biochemical recurrence. The findings indicate that certain radiomics features, such as histogram-based energy and kurtosis features, as well as the shape-based feature representing the standard deviation of the maximum diameter of the prostate gland during treatment, can predict biochemical relapse and identify patients at high risk, concluding that CBCT-based radiomics could be beneficial for treatment definition in PCa.

Delgadillo et al. [83], in a pilot study, investigated the use of CBCT delta-radiomics to predict genitourinary toxicities and International Prostate Symptom Score (IPSS) in PCa patients undergoing radiation therapy. The study used radiomics features extracted from CBCT images and analysed their performance in predicting acute and sub-acute genitourinary toxicities. The study also takes into account the impact of different reconstruction and preprocessing methods on model performance. The results indicate that the radiomics models based on CBCT images have the potential to predict genitourinary toxicities and changes in IPSS, although their performance varied throughout the treatment process.

With this in mind, Mendes et al. [84], discussed the use of radiomics based on CBCT to distinguish between favourable and unfavourable prognosis of PCa. The study evaluated different feature selection methods and classifiers to identify the most effective approach, aiming to provide a tool for monitoring the effectiveness of EBRT for PCa treatment. The results indicate that certain pipelines showed promising performance in distinguishing between favourable and unfavourable cases of PCa, highlighting the potential of CBCT radiomics in evaluating ongoing treatments.

#### 2.2.6. Summary

**Figure 4:**
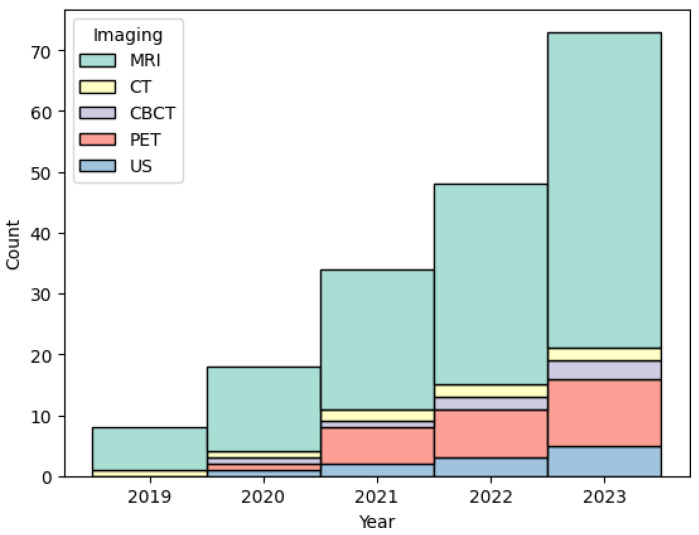
Number of articles per imaging modality and year.

In this review, 55 articles were focused on radiomic features extracted from MRI, the de facto standard for PCa detection and grading. The second most used imaging modality for prostate radiomics is PET (11 studies), followed by US. PET is an important tool for diagnosis, staging, grading and assessing pathological responses of PCa, while US is used in conjunction with prostate biopsy. CBCT (four) and CT (two) are the least used imaging modalities. CT is mainly used for EBRT treatment-planning systems, providing the tissue attenuation coefficients, crucial for dose estimations. CBCT is mainly used for patient setup verification during the EBRT treatment. Still, the results obtained by authors are very promising and may open new opportunities for both imaging modalities to provide insights during EBRT treatments. Figure 4 shows the number of articles by imaging modality and year.

### 2.3. Segmentation

Manual volume delineation is the preferred method performed by highly trained radiation oncologists and medical dosimetrists. Still, it is a time-consuming and error-prone task, and there is always the issue of inter- and intra-observer variability. EBRT Treatment Planning Systems (TPSs) do offer some automated contouring solutions. Most of them are ATLAS-based. A library of images, usually online, with multiple delineated organs of different sizes and shapes, is available to the TPS. These contours are then registered (rigid or elastic) to the patient CT or MRI images. Manual corrections are usually required. EBRT systems also provide tools for manual segmentation along with semi-automatic methods. These can be isodensity threshold or region growing after a manually placed seed point. Additionally, it is possible to propagate the contours to other slices in the volume. The delineated volumes will be the base for radiation dose calculations and optimisations and can be used for radiomic studies.

In this review, 62 studies used manual segmentation, 4 used semi-automatic segmentation and 5 used automatic segmentation. Also, the most used software to perform the segmentation was 3DSlicer [85], a free, open-source software for visualisation, processing, segmentation, registration and analysis of medical and biomedical images. ITK-SNAP [86], also a free, open-source, multi-platform software for application to segment structures in 3D and 4D biomedical images, seems to be a popular option among authors.

### 2.4. Feature Extraction

Feature extraction in radiomics consists of extracting and quantifying image features in a given volume. Combined with other patient information and clinical outcomes, they can provide a potential tool for decision support models [7]. Radiomic features are grouped into two types: semantic and agnostic. Semantic features are qualitative features, often derived from the visual interpretation of medical images by radiologists or clinicians, and include descriptors of tumour shape, size, location, and the presence of specific patterns (e.g., spiculation, necrosis or calcification). Agnostic features are quantitative and are extracted computationally from medical images without a prior hypothesis or specific clinical knowledge guiding the extraction process. The term “agnostic” reflects their extraction and analysis independent of the specific clinical context or human interpretation, relying instead on algorithms to capture various aspects of the image structure and intensity distribution. Agnostic features provide first-order statistics, calculations based on individual voxels reducing the volume to a single value, and second-order descriptors that evaluate the spatial distribution of pixel intensities, capturing the texture and heterogeneity within the image through the analysis of pixel pair relationships and higher-order statistics that search for pattern repetitions in the volume. These can capture more abstract patterns and features not discernible through first- or second-order statistics alone.

**Figure 5:**
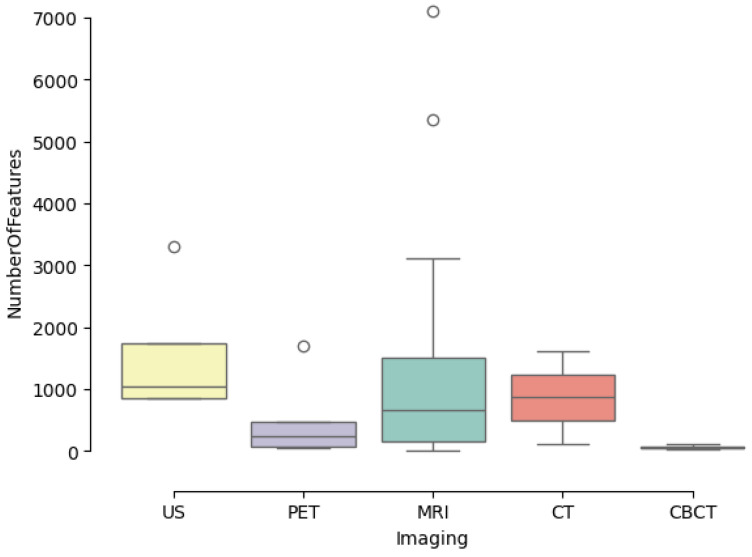
Number of features per imaging modality.

Extracted features must be compliant with the Image Biomarker Standardisation Initiative (IBSI). Figure 5 shows the number of features found in the reviewed papers grouped by imaging modality.

### 2.5. Feature Selection and Model Building

The number of extracted features can increase dramatically in radiomics and even surpass the number of samples, reducing effectiveness and increasing the probability of an overfitting scenario. Before modelling data, feature selection, reduction or recombination should be carried out. There are several commonly used methods for this task, such as Fisher’s discriminant ratio, Principal Component Analysis (PCA), consensus clustering, and others [87]. The main goal is to exclude non-reproducible, redundant and non-relevant features from the dataset [88].

Building the radiomic model usually involves using SVM, decision trees or others. More recently, Convolutional Neural Network (CNN) also contributed to the accelerated progress of radiomics [87]. The best models are those starting with a well-defined endpoint that, in the case of PCa, could be a genomics profile, histology results, serum or biomarkers [7].

The most commonly used feature selection methods by authors in this review are the Least Absolute Shrinkage and Selection Operator (LASSO), Recursive Feature Elimination Support Vector Machine (RFE-SVM) and Minimum-Redundancy-Maximum-Relevance (MRMR). As for the model building techniques, RF, SVM and LR are the preferred ones by most authors.

### 2.6. Best Models

Radiomic studies usually follow a pipeline that involves feature extraction from radiographic images confined to a region of interest, followed by feature selection and model building, as defined by Lambin et al. [6]. Most authors have attempted multiple combinations, searching for the best pipeline. In this section, we present the combinations that provided the best results. Table 1 and Table 2 show the number of features used to build the model, the model-building technique, the clinical endpoint, and the obtained Area Under the Receiver Operating Characteristic (AUROC) value.

## 3. Discussion and Final Remarks

MRI is the preferred imaging method used for radiomic studies of Prostate Cancer. The high spatial resolution makes it highly effective for visualising the prostate anatomy and detecting abnormalities and tumour heterogeneities. The multiple sequences, such as mpMRI, ADC, DWI, and DCE, provide cellular density and vascularity functional information, enhancing the predictive power of radiomic models. Additionally, PIRADS provides guidelines for the acquisition protocols of MRI and a scoring system based on findings and multiple clinical parameters such as PSA level, TNM and GS, widely used in several institutions to establish treatment outcomes.

Despite the prevalence of MRI in radiomic studies for Prostate Cancer, other imaging modalities such as CT, PET or US may offer valuable insights into tumour characterisation and treatment response evaluation. CT is widely available and relatively inexpensive, quick to perform, reducing patient discomfort and motion artefacts. Although CT has inferior soft-tissue contrast to MRI, it can help identify density-related features and structural information, especially when combined with contrast agents.

PET provides metabolic and molecular information, highlighting areas of increased metabolic activity. The extracted features reveal unique functional characteristics, allowing PET to add an extra dimension to radiomic analyses and improving the accuracy of tumour characterisation.

Ultrasound offers real-time imaging, allowing one to perform guided biopsies and other interventional procedures. While not providing the same level of detail as MRI or CT, it can still contribute with valuable texture and shape features, especially in conjunction with contrast agents.

It is also essential to acknowledge the established role of Androgen Deprivation Therapy (ADT) in conjunction with EEBRT for the treatment of Prostate Cancer. ADT has been shown to enhance the efficacy of EBRT by sensitising tumour cells to radiation, thereby improving treatment outcomes. However, hormonal manipulation through ADT can also influence tumour characteristics, potentially altering radiomic features and the interpretation of imaging data.

In summary, integrating features from multiple imaging modalities may enhance the robustness and accuracy of the radiomic model. Standardising acquisition protocols and ensuring high-quality data are also crucial steps in achieving the full potential of radiomics in Prostate Cancer.

Most studies include small and heterogeneous datasets, limiting the robustness and generalisability of the findings. Although some included patients from other institutions and one using a publicly available dataset, most are retrospective and institution-specific, lacking external validation and variability. It may also cause other issues in the dataset, such as imbalanced classes, exclusion of patients with no lesions, no distinction of the peripheral or transitional zone or only pathologically confirmed PCa. Also, demographic and pathophysiological information about the patients is missing in most studies, making any comparison more challenging.

The ground truth is usually manually obtained by one person introducing contouring biasing. TRUS also presents a high false-negative rate and sampling errors. The limitations are several, and some may be hard to overcome to establish radiomic studies as a clinically validated system to aid PCa evaluation. Publicly available datasets can help overcome some of these issues, providing databases with a wide variability of images, segmentation (manual or automatic) and clinical outputs. Also, they can provide a baseline for radiomic features’ reproducibility and generalisability assessment. Greater collaboration between research institutions and the development of shared datasets and methodologies will be crucial in translating the benefits of radiomics into clinical practice.

By addressing these key areas, radiomics can move towards more robust, generalisable, and clinically applicable solutions for Prostate Cancer treatment in EBRT. Future research should focus on collaborative efforts to create large, multicentric datasets and develop standardised methodologies, ensuring the full potential of radiomics in clinical practice. This multidisciplinary and multimodal approach will set the way for more reliable, generalisable, and clinically relevant radiomic models, ultimately improving patient outcomes in Prostate Cancer care.

## Figures and Tables

**Table 1:** Best obtained results (part I).

Imaging	Article	Features	Classifier	Endpoint	AUC
US	Wildeboer et al. [15]	N.A.	RF	csPCa	0.84
	Liang et al. [16]	N.A.	LR	PCa	0.90
	Wang et al. [18]	14	SVM	PCa	0.85
	Qi et al. [19]	13	RF	PCa	0.85
	Sun et al. [17]	20	L1 based	PZ PCa	0.89
PET	Merisaari et al. [20]	2	DA	RG	0.75
	Alongi et al. [21]	N.A.	N.A.	csPCa	0.78
	Cysouw et al. [22]	N.A.	N.A.	Metastatic	0.86
	Erle et al. [23]	77	SVC	Lesion	0.95
	Moazemi et al. [24]	5	SVM	N.A.	0.80
	Papp et al. [25]	N.A.	N.A.	MLH	0.86
	Pirrone et al. [26]	4	N.A.	Response	0.68
	Yao et al. [27]	10	SVM	GS	0.80
	Chan et al. [11]	N.A.	RF	Location	0.95
	Luining et al. [28]	N.A.	RF	LNI	0.88
	Nai et al. [29]	N.A.	KNN	0	0.93
MRI	Abdollahi et al. [30]	N.A.	ADBO	GS	0.78
	Bourbonne et al. [31]	N.A.	N.A.	BCR	0.84
	Chen et al. [32]	10	RF	PCa	1.00
	Min et al. [33]	9	N.A.	csPCa	0.87
	Parra et al. [34]	N.A.	N.A.	csPCa	0.78
	Toivonen et al. [35]	54	LR	GS	0.88
	Varghese et al. [36]	N.A.	QSVM	N.A.	0.92
	Algohary et al. [37]	10	QDA	GG	0.87
	Bernatz et al. [38]	105	RF	csPCa	0.76
	Bleker et al. [39]	N.A.	XGB	csPCa	0.89
	Bourbonne et al. [40]	1	LR	BCR	0.86
	Hou et al. [41]	N.A.	SVM	csPCA	0.89
	Li et al. [42]	N.A.	LR	csPCa	0.98
	Woźnicki et al. [14]	15	N.A.	csPCa	0.89
	Bertelli et al. [44]	N.A.	N.A.	GS	0.80
	Bevilacqua et al. [43]	N.A.	SVM	csPCa	0.84
	Castillo T et al. [46]	N.A.	WORC	GS	0.75
	Castillo T et al. [45]	N.A.	WORC	csPCa	0.91
	Cuocolo et al. [47]	N.A.	SVM	EPE	0.83
	Li et al. [48]	N.A.	Statistical	csPCa	0.91
	Peng et al. [49]	8	SR	csPCa	0.86
	Rodrigues et al. [13]	N.A.	MRMR	csPCa	0.88
	Zhang et al. [50]	8	RF	GS	0.98
	Algohary et al. [51]	N.A.	N.A.	PCa	0.98
	Fan et al. [52]	20	RF	ECE	0.85

**Table 2:** Best obtained results (part II).

Imaging	Article	Features	Classifier	Endpoint	AUC
MRI	Gaudiano et al. [53]	4	N.A.	GG	0.88
	Gresser et al. [55]	N.A.	N.A.	N.A.	0.95
	Jing et al. [56]	10	LR	csPCa	0.97
	Liu et al. [57]	N.A.	RF	0	0.87
	Lu et al. [54]	6	N.A.	PCa	0.87
	Ma et al. [58]	N.A.	LR	GG	0.84
	Sushentsev et al. [59]	N.A.	N.A.	N.A.	0.84
	Yang et al. [12]	N.A.	N.A.	hpIDC-P	0.86
	Dominguez et al. [60]	10	LR	N.A.	0.91
	Gaudiano et al. [61]	4	N.A.	N.A.	0.81
	Isaksson et al. [62]	N.A.	Catboost	Delta T	0.95
	Jamshidi et al. [63]	10	DA	N.A.	0.91
	Jin et al. [64]	N.A.	SVM	csPCa	0.80
	Li et al. [65]	N.A.	N.A.	csPCa	0.96
	Li et al. [66]	N.A.	N.A.	csPCa	0.82
	Liu [67]	6	N.A.	N.A.	0.74
	Midya et al. [68]	N.A.	N.A.	N.A.	0.81
	Prata et al. [69]	1	N.A.	csPCa	0.80
	Qiao et al. [70]	20	LR	GS	0.89
	Qiu et al. [71]	19	N.A.	RG	0.86
	Rodrigues et al. [72]	N.A.	Hybrid	GS	0.87
	Stoyanova et al. [73]	N.A.	N.A.	GG	0.96
	van den Berg et al. [74]	N.A.	LR	EPE	0.91
	Xue et al. [75]	N.A.	N.A.	N.A.	0.93
	Zhao et al. [76]	N.A.	XGBoost	csPCa	0.95
	Zhong et al. [77]	5	RR	N.A.	0.71
	Zhou et al. [78]	15	VM	GS	0.81
CT	Osman et al. [79]	N.A.	N.A.	GS	0.98
	Mendes et al. [80]	N.A.	SVM	RG	0.88
CBCT	Bosetti et al. [82]	3	LR	PSA	0.84
	Delgadillo et al. [83]	N.A.	N.A.	IPSS	0.83
	Mendes et al. [84]	43	SVC	GG	0.82

## Data Availability

Not applicable.

## Abbreviations

The following abbreviations are used in this manuscript:

ADC	Apparent Diffusion Coefficient
ADT	Androgen Deprivation Therapy
AUROC	Area Under the Receiver Operating Characteristic
BCR	Biochemical Recurrence
bmMRI	biparametric MRI
BPH	Benign Prostatic Hyperplasia
CBCT	Cone Beam Computed Tomography
CNN	Convolutional Neural Network
CT	Computed Tomography
DA	Discriminant Analysis
DCE	Dynamic Contrast Enhanced
DWI	Diffusion-weighted Imaging
EBRT	External Beam Radiotherapy
EPE	Extraprostatic Extension
F-18 FDG	Fluorodeoxyglucose
GG	Grade Group
GS	Gleason Score
IBSI	Image Biomarker Standardisation Initiative
ICC	InterCorrelation Coefficient
IMRT	Intensity Modulated Radiation Therapy
IPSS	International Prostate Symptom Score
KNN	K-nearest Neighbors
LASSO	Least Absolute Shrinkage and Selection Operator
LR	Linear Regression
mpMRI	multi-parametric Magnetic Resonance Imaging
MRI	Magnetic Resonance Imaging
MRMR	Minimum-Redundancy-Maximum-Relevance
OAR	Organ At Risk
PCA	Principal Component Analysis
PCa	Prostate Cancer
PET	Positron Emission Tomography
PIRADS	Prostate Imaging Reporting and Data System
PRISMA	Preferred Reporting Items for Systematic Reviews and Meta-Analyses
PSA	Prostate Specific Antigen
PSMA	Prostate-Specific Membrane Antigen
RF	Random Forest
RFE-SVM	Recursive Feature Elimination Support Vector Machine
RG	Risk Group
ROI	Region Of Interest
SUV	Standardized Uptake Value
SVM	Support Vector Machine
TPS	Treatment Planning System
TRUS	Transrectal Ultrasound-Guided Biopsy
US	Ultrasound
